# Effects of Antihypertensive Drugs on Thyroid Function in Type 2 Diabetes Patients With Euthyroidism

**DOI:** 10.3389/fphar.2022.802159

**Published:** 2022-03-07

**Authors:** Lijuan Yang, Xiuqin Sun, Yi Zhao, Hong Tao

**Affiliations:** Department of Endocrinology and Metabolism, Beijing Anzhen Hospital, Capital Medical University, Beijing, China

**Keywords:** thyroid function, type 2 diabetes, thyroid-stimulating hormone, antihypertensive drugs, selective β1-adrenergic receptor blockers

## Abstract

**Objective:** There is little literature about whether antihypertensive drugs would affect thyroid function in patients with euthyroid type 2 diabetes, which was significant in maintaining a proper balance of thyroid function. A retrospective cohort study was conducted to evaluate the influence of antihypertensive drugs on thyroid function in patients with type 2 diabetes with euthyroidism.

**Design and Methods:** The study involved dividing 698 patients with antihypertensive monotherapy into five groups according to the antihypertensive drugs they were treated with. Antihypertensive drugs included in this study were β-blockers, angiotensin-converting enzyme inhibitors (ACEI), angiotensin receptor blockers (ARB), and calcium channel blockers (CCB). The clinical data and thyroid function level between or within groups were compared. Multiple logistic regression analysis was conducted to evaluate the association of antihypertensive drugs with thyroid function level.

**Results:** Selective β_1_- adrenergic receptor blockers treatment was related to thyroid-stimulating hormone (TSH), increasing in patients with diabetes and euthyroidism as shown by multiple logistic regression analysis. The association existed after adjustment for confounding factors. No significant influence on thyroid function was found among other antihypertensive drugs.

**Conclusion:** These data show the TSH-lifting effect of selective β_1_-adrenergic receptor blockers in patients with type 2 diabetes with euthyroidism.

## Introduction

Subjects with type 2 diabetes experience an elevated cardiovascular morbidity and mortality at present, associated with a high prevalence of hypertension, obesity, and dyslipidemia (Punthakee et al., 2007; Zhang et al., 2009). On the other hand, thyroid hormones play a crucial role in regulating many cardiovascular functions (Klein and Ojamaa, 2001). Previous literatures have revealed that decreased endothelial function, high-density lipoprotein cholesterol (HDL-C) level, and increased low-density lipoprotein cholesterol (LDL-C) level, triglycerides (TGs) level, and blood pressure (BP) were observed in persons with higher thyroid-stimulating hormone (TSH) level even when TSH level was still in reference range (Lekakis et al., 1997; Bakker et al., 2001; Asvold et al., 2007a; Asvold et al., 2007b). Therefore, accurately evaluating thyroid function in patients with type 2 diabetes, even when thyroid function was still in normal level, to keep a proper balance of thyroid hormones is necessary (Mullur et al., 2014).

Antidiabetic and antihypertensive treatments are both necessary in diabetes patients with hypertension. As we know, various drugs could influence thyroid function through different mechanisms (Andjelkovic et al., 2016). When evaluating thyroid function, the influence of medication should not be ignored. However, literatures about whether antihypertensive drugs would affect thyroid function were limited, especially in persons with euthyroidism.

Antihypertensive drugs used widely in clinical practice include β-blockers, angiotensin-converting enzyme inhibitors (ACEI), angiotensin receptor blockers (ARB), and calcium channel blockers (CCB) in our hospital. Evidence has linked propranolol therapy to a thyroid function alteration in patients with hyperthyroidism (Faber et al., 1979; Perret et al., 1984; Franklyn et al., 1985) and euthyroidism (Wilkins et al., 1985; Kayser et al., 1991). In recent years, selective β_1_-adrenergic receptor blockers (second-generation agents) and third-generation agents (Rizos et al., 2003; Bakris et al., 2004; Celik et al., 2006) are much more prevalent in clinical practice. However, the evidence for thyroid function-changing effect of second- and third-generation β_1_-adrenergic receptor blockers in euthyroid cases shows contrary outcomes (Kristensen and Weeke, 1977; Faber et al., 1979; Perret et al., 1984; Reeves et al., 1985; Kayser et al., 1991). The effect of ACEI, ARB (Hume et al., 1990; Vranckx et al., 1995; Andjelkovic et al., 2016) and CCB (Brabant et al., 1989; Jangid, 1993; Eidne et al., 1994; Roussel et al., 1995; Roussel et al., 1997; Pedemonte et al., 2005) on thyroid function were not consistent between animal study and clinical studies and the sample size was small (Isles et al., 1985; Haitas et al., 1986; Valensi et al., 1989; Andjelkovic et al., 2016).

Therefore, a clinical study with a larger sample size might be required to investigate whether antihypertensive drugs would affect thyroid function in patients with type 2 diabetes when thyroid function was still in reference range.

## Materials and Methods

### Study Participants

This was a retrospective cohort study examining the effect of antihypertensive therapy on thyroid function in patients with type 2 diabetes and euthyroidism. Patients with diabetes were recruited by searching the computerized database of patients who were treated and followed up at the Department of Endocrinology and Metabolism, Beijing Anzhen Hospital, from December 2014 to December 2020. There were 6,847 type 2 diabetes participants who had at least two visits at the baseline and the end during the 1-year follow-up were included to assess for eligibility. We excluded subjects with the following criteria: 1) without complete personal medical history and detailed drug treatment, missing vital data; 2) treatment with more than one antihypertensive drug during the 1-year follow-up, did not adhere to medication well; 3) thyroid function not within the normal range at baseline, thyroid disease history or therapy; 4) the usage of drugs known to affect thyroid function (metformin, glucocorticoids, dopamine agonists, rexinoids, carbamazepine, metyrapone, etc.) (Cappelli et al., 2012; Andjelkovic et al., 2016); 5) a history of serious cardiovascular events (ischemic heart disease, heart failure, etc.), stroke, chronic obstructive peripheral arteriopathy, gastrointestinal tract disorders (chronic gastritis, *Helicobacter pylori* infection, chronic pancreatitis, and inflammatory bowel disease, etc.), anemia or hemoglobinopathies, malignant disease, liver or renal dysfunction, and a recent history of acute illness; 6) pregnancy; and 7) drug or alcohol abuse.

Type 2 diabetes was diagnosed under World Health Organization (WHO) 1999 criteria (Alberti and Zimmet, 1999), or with a history of type 2 diabetes. Hypertension was defined as systolic blood pressure (SBP) of ≥140 mmHg and/or diastolic blood pressure (DBP) of ≥90 mmHg (Writing Group of Chinese Guidelines for the Management of Hypertension, 2010), or having a history of hypertension. Complications of diabetes and hypertension were recorded as retinopathy complication, kidney disease complication, and peripheral neuropathy complication. Retinopathy complication was determined by fundus examination. Kidney disease complication and peripheral neuropathy complication was defined according to the criteria of Chinese guidelines for the management of hypertension and type 2 diabetes (Writing Group Chinese Guidelines for the Management of Hypertension, 2010; Chinese Diabetes Society of Chinese Medical Association, 2014).

**FIGURE 1:**
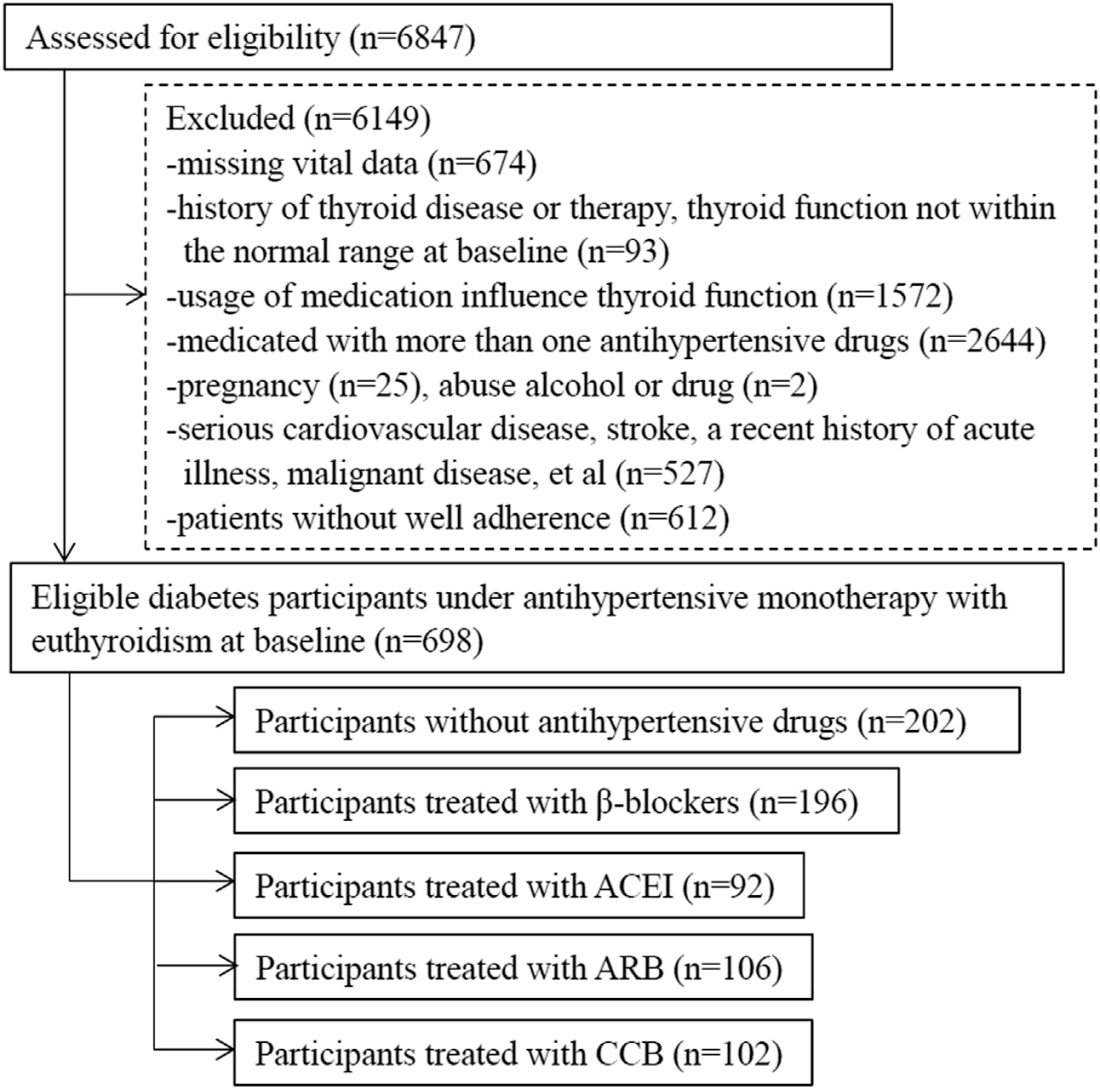
The flowchart of selection.

A total of 698 patients with type 2 diabetes with euthyroidism were collected and divided into five groups based on antihypertensive drugs, including patients without antihypertensive treatment as controls. The process of selection was illustrated in Figure 1.

Group NONE represent group without antihypertensive drug therapy. The medication included in other four groups were metoprolol (47.5 mg, qd), perindopril (4–8 mg, qd), telmisartan (40–80 mg, qd) and nifedipine (30 mg, qd, or 10–20 mg, bid) respectively.

Patients with history of serious cardiovascular events, stroke, chronic obstructive peripheral arteriopathy, et al. often treated with more than one antihypertensive medication. Patients treated with diuretics generally suffered from serious cardiovascular diseases in our hospital and treated with other antihypertensive drug at the same time, such as CCB and ACEI. These patients were not included.

The protocol was conducted in accordance with the Declaration of Helsinki and approved by the Beijing Anzhen Hospital Ethical Committee.

### Clinical Measurements and Biochemical Analysis

The anthropometric indices (height and weight) were measured by professional medical workers using a standard protocol. Weight while wearing light clothing was measured to the nearest 0.1 kg and height to the nearest 1 cm. Body mass index (BMI) was calculated as the body weight (kg) divided by the square of the body height (m). Office BP was measured at the right arm after a minimum of 5 min rest in the sitting position, using an automatic BP monitor (J30 [±3 mmHg], Omron Healthcare Co., Ltd., Kyoto, Japan), and the average of two measurements were recorded. The sociodemographic characteristics, including age, sex, smoking status, alcohol consumption, individual history of diseases (including cardiovascular disease (CVD), hypertension, dyslipidemia, type 2 diabetes, complications of diabetes and hypertension, and thyroid disorders), and detailed drug treatment, were also collected from participants’ medical records.

The venous blood samples were drawn between 6 after midnight (AM) to 8 AM from overnight fasting at least 10 h subjects for the measurement of thyroid function, serum lipid and renal function. Serum concentrations of TSH (third-generation TSH assay; normal range: 0.49–4.91 mIU/L), free triiodothyronine (fT3; normal range: 3.28–6.47 pmol/L), free thyroxine (fT4; normal range: 7.64–16.03 pmol/L), antithyroglobulin antibody (TgAb, normal range: 0–4.9 IU/ml) and thyroperoxidases antibody (TPOAb, normal range: 0–9 IU/ml) were measured using the Chemi-Luminescence method by an automatic analyzer (UniCel DxI800 Access Immunoassay System, BECKMAN COULTER, United States) using reagents from BECKMAN COULTER, Inc. Serum total cholesterol (TC), LDL-C, HDL-C, and TGs were measured by colorimetry methods. Creatinine (Cr) was measured by picric acid method. Fasting blood glucose (FBG) was measured using hexose kinase method. These measurements were performed on an automated instrument (Cobas 8000; Roche Diagnostics GmbH, Mannheim, Germany) using reagents from Roche diagnostics. Hemoglobin A1c (HbA1c) was measured using high performance liquid chromatography by HbA1c analyzer (Premier Hb9210, Primus Corporation, United States) using reagents from Trinity Biotech. The intra- and interassay coefficients of variation were below 5% for the above parameters. Jostel’s TSH index (TSHI) and thyroid’s secretory capacity (SPINA-GT) were calculated using the equation described by Dietrich et al. (2016).

### Statistical Analysis

The Shapiro–Wilk test was used to assess the distributional characteristic of variables. For continuous variables, data with normal distribution or approximate normal distribution were expressed with means and standard deviations (SD), or in the case of non-normal distribution, with medians and interquartile ranges (IQR). Categorical variables were recorded as absolute numbers with percentages.

Analysis of variance (ANOVA) followed by *post hoc* analysis of significance (Bonferroni’s test) for continuous variables and Pearson’s χ^2^ test for categorical variables were used to compare clinical data among different categories of antihypertensive therapy. A Welch test was used when the data were non-constant variances in ANOVA. For data with non-normal distribution, a nonparametric test (Kruskal Wallis test followed by Bonferroni’s test) was conducted. The measurements comparison between baseline and at the end of the study was performed by a paired student *t*-test, and Wilcoxon’s Sign Rank Test was used for non-normal distribution data. D-values of data between the end of the study and baseline were calculated and the distribution was assessed. When comparing thyroid hormone level change under different antihypertensive medication, ANOVA and paired student *t*-test were both used and D-value was evaluated further.

Multiple logistic regression models with thyroid function levels at 1 year of medication as the dependent variable were used to identify the relationship between the thyroid function levels and the independent variables, including different antihypertensive therapy, gender, age, BMI, BP, smoking, alcohol consumption, diabetes/hypertension duration, HbA1c, serum lipid profile, estimated glomerular filtration rate (eGFR), thyroid autoimmunity, statin therapy, antidiabetes medicine, and complications of diabetes and hypertension. These were expressed as odds ratios (ORs) with 95% confidence intervals (95% CIs). The continuous variable of thyroid function level was transferred to a categorical variable. A dummy variable was set for transforming the categorical variable of antihypertensive medications, and the no antihypertensive treatment group was identified as control.

A *p*-value of less than 0.05 was regarded as statistically significant. We used Statistical Package for Social Sciences version 23.0 (SPSS Inc., ver.23, Chicago, IL, United States) for calculations and statistical analyses.

## Results

### Participant Characteristics

Clinical data of patients subdivided into the five groups according to their drug therapy at recruitment are shown in Table 1. The differences in gender, age, BMI, cigarette smokers, and alcohol drinkers were not significant (*p* > 0.05) among the study groups. Serum TSH, fT_3_, fT_4_, thyroid antibodies, serum lipid profile, FBG, HbA1c, Cr, and eGFR were superimposable among the five groups (*p* > 0.05). All patients maintained diet-treatment and moderate activities. The percentages of antidiabetes and statins therapy, complications of diabetes and hypertension, duration of diabetes were comparable among the groups (*p* > 0.05). DBP and SBP was higher in antihypertensive treatment groups (*p* < 0.05), as well as the duration of hypertension was longer in antihypertensive treatment groups (*p* < 0.05), and no significant difference of DBP, SBP and duration of hypertension was found among the four antihypertensive treatment groups (*p* > 0.05).

**TABLE 1:** Clinical data of patients grouped according to their drug therapy at recruitment.

	None	β-blockers	ACEI	ARB	CCB	*p* value
Patients (*n*)	202	196	92	106	102	
Gender (*n*, %)	90 (44.6%)	98 (50.0%)	51 (55.4%)	39 (36.8%)	51 (50.0%)	0.071
Age (years)	61.0 ± 11.7	63.5 ± 9.9	62.0 ± 12.0	63.2 ± 13.0	61.0 ± 12.5	0.135^a^
BMI (kg/m^2^)	25.710 ± 3.373	25.856 ± 3.210	26.418 ± 3.037	26.397 ± 3.260	26.310 ± 3.155	0.201
SBP (mmHg)	128.376 ± 13.355	138.464 ± 12.223	136.728 ± 11.731	137.943 ± 13.202	136.069 ± 13.434	0.000^b^
DBP (mmHg)	78.614 ± 9.774	83.709 ± 9.317	84.293 ± 11.084	85.113 ± 10.794	86.029 ± 10.006	0.000^b^
DM duration (years)	4.0 (6.0)	3.0 (7.0)	5.0 (9.0)	5.0 (9.0)	5.0 (8.3)	0.087^c^
HT duration (years)	4.0 (5.0)	6.0 (7.0)	6.0 (13.8)	7.0 (10.3)	6.0 (8.0)	0.000^d^
TSH (mIU/L)	2.304 ± 0.902	2.333 ± 0.950	2.332 ± 1.023	2.313 ± 0.938	2.394 ± 0.931	0.956
fT_3_ (pmol/L)	4.856 ± 0.602	4.874 ± 0.654	4.965 ± 0.641	4.782 ± 0.585	4.833 ± 0.632	0.341
fT_4_ (pmol/L)	11.053 ± 1.781	11.366 ± 1.767	11.286 ± 1.655	11.336 ± 1.433	11.198 ± 1.798	0.416
TPOAb/TgAb(+) (*n*, %)	18 (8.9%)	18 (9.2%)	8 (8.7%)	11 (10.4%)	9 (8.8%)	0.993
TC (mmol/L)	4.783 ± 1.381	4.544 ± 1.215	4.574 ± 1.247	4.509 ± 0.989	4.607 ± 0.962	0.304^a^
HDL-C (mmol/L)	1.249 ± 0.355	1.245 ± 0.347	1.184 ± 0.295	1.272 ± 0.342	1.246 ± 0.287	0.447
LDL-C (mmol/L)	2.791 ± 1.004	2.638 ± 0.996	2.617 ± 1.054	2.621 ± 0.764	2.680 ± 0.833	0.441^a^
TGs (mmol/L)	1.897 ± 1.914	1.759 ± 1.094	2.077 ± 1.636	1.821 ± 1.402	1.929 ± 1.556	0.566
FBG (mmol/L)	7.563 ± 2.416	7.501 ± 2.461	8.004 ± 2.491	7.966 ± 2.783	7.836 ± 3.050	0.360
HbA1c (%)	6.839 ± 1.453	6.874 ± 1.445	7.034 ± 1.426	7.004 ± 1.322	7.145 ± 1.773	0.426
Cr (umol/L)	67.513 ± 15.026	69.899 ± 14.578	70.829 ± 17.714	68.724 ± 15.038	69.186 ± 15.101	0.414
eGFR (mL/min/1.73 m^2^)	93.730 ± 16.070	89.811 ± 14.208	91.414 ± 17.047	90.004 ± 16.490	93.053 ± 14.723	0.080
Statin treatment (*n*, %)	90 (44.6%)	96 (49.0%)	43 (46.7%)	48 (45.3%)	52 (51.0%)	0.815
Glucosidase inhibitors (*n*, %)	92 (45.5%)	94 (48.0%)	43 (46.7%)	58 (54.7%)	44 (43.1%)	0.503
Sulfonylureas (*n*, %)	60 (29.7%)	67 (34.2%)	29 (31.5%)	34 (32.1%)	38 (37.3%)	0.725
TZD (*n*, %)	45 (22.3%)	40 (20.4%)	15 (16.3%)	17 (16.0%)	18 (17.6%)	0.604
DPP-IV inhibitors (*n*, %)	53 (26.2%)	47 (24.0%)	19 (20.7%)	27 (25.5%)	25 (24.5%)	0.886
SGLT-2 inhibitors (*n*, %)	41 (20.3%)	36 (18.4%)	17 (18.5%)	28 (26.4%)	20 (19.6%)	0.539
Insulin (*n*, %)	78 (38.6%)	77 (39.3%)	43 (46.7%)	46 (43.4%)	41 (40.2%)	0.692
GLP-1 ra (*n*, %)	42 (20.8%)	44 (22.4%)	13 (14.1%)	25 (23.6%)	20 (19.6%)	0.493
Retinopathy (*n*, %)	85 (42.1%)	95 (48.5%)	45 (48.9%)	53 (50.0%)	45 (44.1%)	0.583
Kidney disease (*n*, %)	85 (42.1%)	92 (46.9%)	46 (50.0%)	54 (50.9%)	50 (49.0%)	0.538
Peripheral neuropathy (*n*, %)	91 (45.0%)	96 (49.0%)	44 (47.8%)	55 (51.9%)	45 (44.1%)	0.746
Smokers (*n*, %)	74 (36.6%)	67 (34.2%)	24 (26.1%)	30 (28.3%)	37 (36.3%)	0.304
Drinkers (*n*, %)	29 (14.4%)	30 (15.3%)	13 (14.1%)	18 (17.0%)	22 (21.6%)	0.535

Data with normal distribution or approximate normal distribution were expressed as mean ± SD. Data with non-normal distribution were expressed with medians and interquartile ranges (IQR). Categorical variables were recorded as absolute numbers with percentages. Retinopathy, kidney disease and peripheral neuropathy represent complications of diabetes and hypertension. TPOAb/TgAb(+) was defined as serum TPOAb/TgAb higher than their upper limit of reference ranges. β-blockers, selective β_1_-adrenergic receptor blockers; ACEI, angiotensin-converting enzyme inhibitors; ARB, angiotensin receptor blockers; CCB, calcium channel blockers; BMI, body mass index; SBP, systolic blood pressure; DBP, diastolic blood pressure; DM, diabetes mellitus; HT, hypertension; TSH, thyroid-stimulating hormone; fT3, free triiodothyronine; fT4, free thyroxine; TPOAb, thyroperoxidases antibody; TgAb, antithyroglobulin antibody; TC, total cholesterol; HDL-C, high-density lipoprotein cholesterol; LDL-C, low-density lipoprotein cholesterol; TGs, triglycerides; FBG, fasting blood glucose; HbA1c, hemoglobin A1c; Cr, creatinine; eGFR, estimated glomerular filtration rate; TZD, thiazolidinediones; DPP-IV inhibitors, dipeptidyl peptidase-IV inhibitors; SGLT-2 inhibitors, sodium-dependent glucose transporters-2 inhibitors; GLP-1 ra, glucagon likepeptide-1 receptor agonists.

a Welch test is used for non-constant variance.

b Bonferroni’s test revealed significant difference in SBP/DBP between patients without antihypertensive medication and other four groups, and no significance difference among the four groups with antihypertensive medication.

c Kruskal Wallis test is used for non-normal distribution variance.

d Kruskal Wallis test followed by Bonferroni’s test for non-normal distribution variance revealed significant difference in HT duration between patients without antihypertensive medication and other four groups, and no significance difference among the four groups with antihypertensive medication.

The characteristics of subjects in the five groups at 1-year follow-up were provided in Table 2. There was no significant difference in index among the five groups, including SBP and DBP (*p* > 0.05). Positive TPOAb/TgAb did not change after treatment.

**TABLE 2:** Clinical data of patients at 1-year follow-up.

	None	β-blockers	ACEI	ARB	CCB	*p* value
Patients (*n*)	202	196	92	106	102	
BMI (kg/m^2^)	25.825 ± 3.220	25.952 ± 3.086	26.168 ± 2.896	26.304 ± 3.040	26.206 ± 2.986	0.667
SBP (mmHg)	128.980 ± 10.665	130.582 ± 9.185	129.565 ± 9.321	130.368 ± 10.166	128.235 ± 9.412	0.253
DBP (mmHg)	80.252 ± 6.453	79.571 ± 7.065	81.315 ± 6.709	80.717 ± 7.308	81.235 ± 6.774	0.175
TC (mmol/L)	4.374 ± 1.092	4.214 ± 1.059	4.221 ± 1.099	4.394 ± 0.829	4.240 ± 0.957	0.356^a^
HDL-C (mmol/L)	1.263 ± 0.353	1.211 ± 0.354	1.214 ± 0.252	1.264 ± 0.286	1.188 ± 0.277	0.170^a^
LDL-C (mmol/L)	2.535 ± 0.838	2.389 ± 0.818	2.390 ± 0.887	2.516 ± 0.642	2.441 ± 0.774	0.344^a^
TGs (mmol/L)	1.589 ± 0.921	1.787 ± 1.381	1.937 ± 1.793	1.692 ± 1.178	1.753 ± 1.027	0.233
FBG (mmol/L)	6.996 ± 1.681	7.448 ± 2.030	7.415 ± 1.665	7.167 ± 1.609	7.097 ± 1.933	0.100
HbA1c (%)	6.620 ± 0.999	6.825 ± 1.298	6.952 ± 1.047	6.770 ± 1.014	6.811 ± 1.247	0.168
Cr (umol/L)	69.282 ± 13.668	71.643 ± 14.042	72.411 ± 16.005	70.545 ± 13.514	72.416 ± 15.916	0.266
eGFR (mL/min/1.73 m^2^)	91.596 ± 16.017	88.180 ± 14.991	89.787 ± 16.441	88.293 ± 16.476	89.485 ± 16.376	0.244

Data with normal distribution or approximate normal distribution were expressed as mean ± SD. β-blockers, selective β_1_-adrenergic receptor blockers; ACEI, angiotensin-converting enzyme inhibitors; ARB, angiotensin receptor blockers; CCB, calcium channel blockers; BMI, body mass index; SBP, systolic blood pressure; DBP, diastolic blood pressure; TC, total cholesterol; HDL-C, high-density lipoprotein cholesterol; LDL-C, low-density lipoprotein cholesterol; TGs, triglycerides; FBG, fasting blood glucose; HbA1c, hemoglobin A1c; Cr, creatinine; eGFR, estimated glomerular filtration rate.

a Welch test is used for non-constant variance.

### Changes in Thyroid Function Among Participants Grouped According to Their Medication

TSH levels increased only in patients undergoing metoprolol treatment and reached statistical significance (from 2.333 ± 0.950 to 2.993 ± 0.956 mIU/L, *p* < 0.001). These data show that TSH serum levels were significantly higher in the group of patients treated with selective β_1_-adrenergic receptor blockers compared with all other groups at the end of the study (*F* = 16.176, *p* < 0.001). The values of TSH level did not differ among the other therapy groups (Table 3). The level of serum fT_3_ under the treatment of metoprolol decreased, but this was not statistically significant (from 4.874 ± 0.654 to 4.770 ± 0.680 pmol/L, *p* = 0.059) (Table 4). Serum fT_4_ levels did not change significantly in any group from baseline to the end of the study (Table 5).

**TABLE 3:** TSH levels at baseline and at the end of the study in each group (mIU/L).

	Baseline	After medication	*t* value	*p* value	D-value (after medication-baseline)
None	2.304 ± 0.902	2.296 ± 0.941	0.195	0.846	−0.008 ± 0.568
β-blockers	2.333 ± 0.950	2.993 ± 0.956^a^	−13.626	0.000	0.661 ± 0.679^b^
ACEI	2.332 ± 1.023	2.380 ± 1.023	−0.812	0.419	0.048 ± 0.565
ARB	2.313 ± 0.938	2.399 ± 0.922	−1.625	0.107	0.086 ± 0.542
CCB	2.394 ± 0.931	2.389 ± 0.954	0.094	0.925	−0.005 ± 0.547
*F* value	0.166	16.176			41.103
*p* value	0.956	0.000			0.000

Data are presented as mean ± SD. ACEI, angiotensin-converting enzyme inhibitors; ARB, angiotensin receptor blockers; CCB, calcium channel blockers; TSH, thyroid-stimulating hormone.

a Bonferroni’s test revealed significant difference in TSH level between patients medication with β-blockers and other medication.

b Bonferroni’s test revealed significant difference in D-value (after medication-baseline) between patients medication with β-blockers and other medication.

**TABLE 4:** FT3 levels at baseline and at the end of the study in each group (pmol/L).

	Baseline	After medication	*t* value	*p* value	D-value (after medication-baseline)
None	4.856 ± 0.602	4.881 ± 0.571	−0.684	0.495	0.025 ± 0.523
β-blockers	4.874 ± 0.654	4.770 ± 0.680	1.902	0.059	−0.104 ± 0.766
ACEI	4.965 ± 0.641	4.913 ± 0.603	1.129	0.262	−0.051 ± 0.435
ARB	4.782 ± 0.585	4.807 ± 0.604	−0.356	0.723	0.024 ± 0.701
CCB	4.833 ± 0.632	4.700 ± 0.622	1.699	0.092	−0.133 ± 0.790
*F* value	1.130	2.290			1.712
*p* value	0.341	0.058			0.145

Data are presented as mean ± SD. ACEI, angiotensin-converting enzyme inhibitors; ARB, angiotensin receptor blockers; CCB, calcium channel blockers; fT3, free triiodothyronine.

**TABLE 5:** FT4 levels at baseline and at the end of the study in each group (pmol/L).

	Baseline	After medication	*t* value	*p* value	D-value (after medication-baseline)
None	11.053 ± 1.781	11.307 ± 1.653	−1.885	0.061	0.254 ± 1.918
β-blockers	11.366 ± 1.767	11.166 ± 1.719	1.369	0.173	−0.201 ± 2.053
ACEI	11.286 ± 1.655	11.579 ± 1.811	−1.339	0.184	0.293 ± 2.098
ARB	11.336 ± 1.433	11.159 ± 1.544	0.979	0.330	−0.177 ± 1.865
CCB	11.198 ± 1.798	11.377 ± 1.872	−0.865	0.389	0.180 ± 2.100
*F* value	0.983	1.142			2.085
*p* value	0.416	0.335			0.081

Data are presented as mean ± SD. ACEI, angiotensin-converting enzyme inhibitors; ARB, angiotensin receptor blockers; CCB, calcium channel blockers; fT4, free thyroxine.

Then we proceeded to evaluate pituitary thyrotropic function indice TSHI, and the structure parameter of thyroid homeostasis SPINA-GT. The value of TSHI and SPINA-GT after and before β_1_-blockers therapy were (2.538 ± 0.428 vs 2.272 ± 0.493) and (1.625 (0.559) pmol/s vs 1.861 (0.964) pmol/s), respectively. A significant increase and decrease was found in TSHI (*t* = −9.119, *p* < 0.001) and SPINA-GT (*z* = 8.125, *p* < 0.001) after β-blocker therapy, respectively.

### Influencing Factors

In order to conduct multiple logistic regression analysis, the continuous variable of D-value of TSH levels was transferred to a categorical variable. D-value was calculated by TSH level of after medication minus at baseline. We defined |D-value| ≥0.5 mIU/L as a marked change. Pearson’s χ^2^ test followed by *post hoc* analysis of significance (Bonferroni’s test) revealed a statistical significance of proportion of D-value with marked change under medication of selective β_1_-adrenergic receptor blockers and other treatment (*p* < 0.001). Proportion of D-value with marked change of other medications did not differ significantly (Table 6). This result was consistent with ANOVA analysis (Table 3) shown above.

**TABLE 6:** D-value (categorical variable transferred) of TSH level among each group.

	None	β-blockers	ACEI	ARB	CCB	*F/p* value
Marked change	63 (31.2%)	129 (65.8%)^a^	34 (37.0%)	32 (30.2%)	28 (27.5%)	*F* = 71.437
Without marked change	139 (68.8%)	67 (34.2%)^a^	58 (63.0%)	74 (69.8%)	74 (72.5%)	*p* = 0.000

Data are presented as absolute numbers with percentages. ACEI, angiotensin-converting enzyme inhibitors; ARB, angiotensin receptor blockers; CCB, calcium channel blockers; TSH, thyroid-stimulating hormone.

a Bonferroni’s test revealed that the difference between prevalence of D-value with marked change under medication of selective β-blockers and other treatment was statistical significant.

The results of multiple logistic regression analysis with subjects with/without D-value of TSH with marked change as dependent variables, and antihypertensive drugs, gender, age, BMI, BP, smoking, alcohol consumption, diabetes/hypertension duration, serum lipid profile, HbA1c, eGFR, thyroid autoimmunity, statin therapy, antidiabetes medicine, and complications of diabetes and hypertension at baseline as independent variables, are shown in Table 7. Compared with the non-antihypertensive treatment group, the effect on TSH levels under the usage of selective β_1_-adrenergic receptor blockers was statistically significant (*OR* = 4.147, 95% CI 2.617–6.572, *p* < 0.001) among the four antihypertensive drugs. Other confounding factors were not significant variables in this model.

**TABLE 7:** Multiple logistic regression analysis for prevalence of D-value of TSH with marked change as dependent variable in the whole group (*n* = 698).

Variable	*B*	*EXP(B)*	95% CI of *EXP(B)*	*p* value
Gender	0.032	1.032	0.716–1.489	0.865
Age	−0.018	0.982	0.963–1.001	0.070
BMI	−0.028	0.973	0.924–1.024	0.290
SBP	0.007	1.007	0.993–1.020	0.339
DBP	0.001	1.001	0.983–1.019	0.935
DM duration	0.013	1.013	0.979–1.048	0.468
HT duration	0.015	1.015	0.988–1.042	0.289
TC	0.072	1.074	0.752–1.536	0.694
HDL-C	0.033	1.033	0.511–2.090	0.928
LDL-C	0.037	1.038	0.704–1.530	0.851
TGs	−0.047	0.954	0.794–1.147	0.618
HbA1c	−0.125	0.883	0.778–1.001	0.053
eGFR	−0.003	0.997	0.983–1.010	0.637
TPOAb/TgAb(+)	−0.353	0.703	0.389–1.270	0.243
Selective β-blockers	1.422	4.147	2.617–6.572	0.000
ACEI	0.093	1.097	0.620–1.941	0.750
ARB	−0.148	0.863	0.490–1.518	0.609
CCB	−0.322	0.725	0.408–1.287	0.272
Statins treatment	0.311	1.364	0.971–1.916	0.073
Glucosidase inhibitors	-0.211	0.810	0.563–1.165	0.255
Sulfonylureas	0.007	1.007	0.683–1.485	0.972
Thiazolidinediones	−0.208	0.812	0.524–1.259	0.352
DPP-IV inhibitors	−0.363	0.696	0.454–1.066	0.096
SGLT-2 inhibitors	0.042	1.043	0.687–1.582	0.844
Insulin	0.091	1.095	0.731–1.639	0.659
GLP-1 receptor agonists	−0.332	0.718	0.459–1.122	0.146
Retinopathy	−0.078	0.925	0.420–2.038	0.846
Kidney disease	0.273	1.314	0.579–2.984	0.513
Peripheral neuropathy	−0.131	0.878	0.535–1.441	0.606
Smokers	−0.299	0.741	0.501–1.097	0.134
Drinkers	0.246	1.279	0.779–2.099	0.330

Retinopathy, kidney disease and peripheral neuropathy represent complications of diabetes and hypertension. TPOAb/TgAb(+) was defined as serum TPOAb/TgAb higher than their upper limit of reference ranges. TSH, thyroid-stimulating hormone; BMI, body mass index; SBP, systolic blood pressure; DBP, diastolic blood pressure; DM, diabetes mellitus; HT, hypertension; TC, total cholesterol; HDL-C, high-density lipoprotein cholesterol; LDL-C, low-density lipoprotein cholesterol; TGs, triglycerides; HbA1c, hemoglobin A1c; eGFR, estimated glomerular filtration rate; TPOAb, thyroperoxidases antibody; TgAb, antithyroglobulin antibody; ACEI, angiotensin-converting enzyme inhibitors; ARB, angiotensin receptor blockers; CCB, calcium channel blockers; DPP-IV inhibitors, dipeptidyl peptidase-IV inhibitors; SGLT-2 inhibitors, sodium-dependent glucose transporters-2 inhibitors; GLP-1 receptor agonists, glucagon likepeptide-1 receptor agonists.

### Changes in Thyroid Function in Participants Whose Selective β_1_- Adrenergic Receptor Blockers Were Discontinued After One Year’s Medication

In screening the computerized database, we found selective β_1_-adrenergic receptor blockers were discontinued in 11 patients of group β-blockers after the elevation of TSH levels, for the following reasons: 1) heart rate ≤55 beats per minute (2/11); 2) medication treating hypertension was changed from β-blockers to CCB (3/11); 3) medication treating hypertension was changed from β-blockers to ACEI (2/11); 4) exact reason not found (4/11). Seven men and four women were included. The doses of metoprolol medication of the 11 patients were 47.5 mg/d. D-value of TSH level was calculated, and the distribution was assessed. A paired student *t*-test was conducted to evaluate TSH level changes. After 1 year’s medication of selective β-blockers, TSH levels of the 11 patients were (2.659 ± 1.460) mIU/L, a significant increase compared with the base level (2.659 ± 1.460 vs. 1.814 ± 1.293 mIU/L, D-value = 0.846, 95% CI 0.489–1.202, *p* = 0.000). The period without metoprolol medication varied from three to 17 months. After withdrawal of metoprolol, TSH value dropped to the level close to baseline value at the beginning (2.066 ± 1.426 vs, 1.814 ± 1.293 mIU/L, D-value = 0.252, 95% CI −0.013 to 0.517, *p* = 0.060).

## Discussion

The results of the retrospective study herein showed the TSH-lifting effect of selective β_1_-adrenergic receptor blockers, whereas no significant influence was found among other antihypertensive drugs. No matter whether we adjusted confounding factors or not, the association between β-blockers and thyroid functions existed. As far as we know, this was the first study to assess the association between thyroid function and antihypertensive drugs in patients with type 2 diabetes with euthyroidism.

β-adrenergic receptor blocking drugs have been widely used in clinical practice for more than 50 years (Frishman, 2008). Besides the β-blocking activity of this agent, another mechanism was the influence on the peripheral metabolism of circulating thyroid hormone. Studies consistently reported that β-adrenergic receptor blocking drugs decreases serum triiodothyronine (T_3_) in patients with hyperthyroidism (Faber et al., 1979; Perret et al., 1984; Franklyn et al., 1985). However, similar serum T_3_-lowering effects in patients with euthyroidism were inconsistent (Kristensen and Weeke, 1977; Perret et al., 1984; Franklyn et al., 1985; Wilkins et al., 1985; Kayser et al., 1991). There are also conflicting reports regarding the influence of β-adrenergic receptor blocking drugs on TSH levels in euthyroid patients (Kristensen and Weeke, 1977; Faber et al., 1979; Perret et al., 1984; Reeves et al., 1985; Kayser et al., 1991). Perret et al. (1984) reported that TSH concentrations remained stable under treatment with propranolol and acebutolol, which was in accordance with studies done by Faber et al. (1979) (propranolol), Kristensen and Weeke (1977) (propranolol), and Reeves et al. (1985) (propranolol and nadolol). But Kayser et al. have demonstrated that after a 1-week therapy of propranolol and atenolol, median serum TSH levels increased, whereas median serum TSH decreased compared with pretreatment values after 3 weeks of therapy. However, the increase of TSH levels under the treatment of metoprolol did not reach statistical significance (Kayser et al., 1991).

In this study, performed on larger sample size, we showed that treatment with metoprolol is associated with a significant rise in the serum levels of TSH in patients with euthyroid type 2 diabetes. The design of the current study was not aimed at a possible explanatory mechanism for the TSH-lifting effect of β-adrenergic receptor blocking drugs. The possible mechanisms from previous literatures were as followed. First, the regulatory β_1_-adrenergic receptors normally mediated the inhibitory effect of epinephrine and norepinephrine on the TSH secretion from the pituitary gland (Kayser et al., 1991). Blocking of the regulatory β_1_-adrenergic receptors may cause an increase in serum TSH levels. But Melander et al. (1977) reported that adrenergic terminals had not been found in the hypothalamus or the pituitary of the mouse. In the current study, TSHI lifted under the treatment of β_1_-adrenergic blockers might indicate an increase in TSH secretion. Our result required more verify. Second, the changes in the peripheral thyroxine (T_4_) and T_3_ values could influence serum TSH levels in negative feedback mode. In Kuntz’s literature, repeated proofs had indicated that thyroid gland function could be adjusted by sympathetic and parasympathetic nerves, and the nerve fibers terminate enter the thyroid gland might were distributed mainly to the thyroid follicles and the walls of the blood vessels (Kuntz, 1932). Melander et al. (1977) reported that the sympathetic nerve system could exert a direct, stimulatory effect on the secretion of the thyroid hormone, independently from TSH levels. However Hotta et al. (2017) discovered that the secretion of thyroid hormone increased during parasympathetic (superior laryngeal nerve) stimulation and decreased during sympathetic (cervical sympathetic trunk) stimulation, this result might associated with various nerve fibers were contained in superior laryngeal nerve, and further investigation was necessary. Our investigation showed SPINA-GT decreased by the sympathetic activity blocking effect under β-blockers treatment, which was consistent with Melander’s animals study (Melander et al., 1977) and the mechanism of β-blockers, indicating the decreased secretion of thyroid hormone under β-blockers. Third, sympathetic nervous system may exert a stimulatory influence on the deiodination of thyroid hormone (Melander et al., 1977). Several studies supported that β-adrenergic receptor blockers decrease serum T_3_ due to inhibition of the conversion of T_4_ into T_3_ and provided evidence for an inhibitory influence of the type 1 or 2 deiodinase enzyme (Perrild et al., 1983; Wilkins et al., 1985; Wiersinga, 1991). This enzyme is responsible for the deiodinase from T_4_ to T_3_ (Chopra et al., 1978). The mechanism by which β-adrenergic receptor blockers exert this inhibition on the deiodinase enzyme is unclear. In the current study, a slight fall in serum T_3_ was found but was not statistically significant. But what should not be ignored was that the inhibition effect of the deiodinase enzyme was studied most on propranolol (unselective β-adrenergic receptor blockers). Our investigation was made on metoprolol, selective β-adrenergic receptor blockers, and the proof about inhibition effect by metoprolol was limited that was reported by Perrild’ study (Perrild et al., 1983), which need more to confirm. As reported by Carr et al. (1988), even small alterations of thyroxine dosages given to patients with primary hypothyroidism could cause basal TSH values to change. Therefore, alterations of TSH level mediated by T_4_ or T_3_ value could not be excluded.

Above all, the effect that selective β_1_-adrenergic receptor blockers may result in a rise in serum TSH levels is biologically reasonable, but studies to investigate the mechanisms are required.

A possible reason why no significant decrease in T_3_ and T4 level was found could be related to the medicine dose. In Wilkins’s study (Wilkins et al., 1985), a significant fall of T_3_ level emerged with a higher propranolol dose. In this study, a relatively lower dose of metoprolol probably could not exert enough inhibitory influence to produce a significant fall of T_3_ and T4 level.

No significant variation was observed in serum TSH and thyroid hormone levels under the treatment of ACEI, ARB, and CCB. Renin-angiotensin-aldosterone system (RAAS) is activated by thyroid hormones in hyperthyroidism (Silverstein et al., 1983). But no association between ACEI and ARB treatment with thyroid function change was found with euthyroid ones in this study, in accordance with other clinical studies (Hume et al., 1990; Andjelkovic et al., 2016), conflicting with an animal study (Vranckx et al., 1995). The increased intracellular availability of Ca^2+^ plays an important role in the regulation and secretion of thyroid hormones (Lalau et al., 1987). Studies indicated that CCB could interfere with thyroid function through pituitary (Brabant et al., 1989; Roussel et al., 1995; Roussel et al., 1997), thyroid (Jangid, 1993; Pedemonte et al., 2005), and peripheral thyroid physiological process (Capen, 1994). However, the clinical studies on the effect of CCB on thyroid function were inconsistent (Isles et al., 1985; Haitas et al., 1986; Valensi et al., 1989). The probable mechanism there was no effect on thyroid function from CCB in the current study might was another Ca^2+^ pathway existing could not be prevented by CCB (Corda et al., 1985) and oral treatment (Semple et al., 1984).

Previous study showed that statins use was associated with lower TSH levels and the relationship was mediated by TC declines (Wang et al., 2021). Our investigation did not found this association might because TC reduction was not strong enough, which was partly consistent with Robert’s study (Krysiak et al., 2019), or other hidden reason. Specially designed prospective study might be needed.

Decreased endothelial function, HDL-C level, and increased LDL-C level, TGs level, and BP were associated with higher TSH level even when TSH level was in normal range (Lekakis et al., 1997; Bakker et al., 2001; Asvold et al., 2007a; Asvold et al., 2007b), companied with a negative consequence on glucose metabolism by β-blockers treatment (Jacob et al., 1996), which remind clinical medical workers to pay attention to TSH-lifting effect in euthyroid diabetes patients treated with β-blockers. On the other hand, one of the most important therapeutic utility on non-cardiovascular diseases by β-blockers was on hyperthyroidism and thyroid storm. Except the blocking effect on sympathetic over-activity and of thyroid hormones in the cardiovascular system, the possible inhibitory effect on secretion of thyroid hormone by β-blockers may benefit the prognosis of hyperthyroidism and thyroid storm, which deserved more attention and required further confirmation.

This study has several limitations. First, this was a retrospective study. The most important criterion was to ensure the participants were under antihypertensive monotherapy throughout the follow-up. We tried to overcome this point by repeatedly confirming the medication with patients and excluded those without well adherence. However, we cannot be sure that there were no patients who did not follow the doctor’s advice. Another disadvantage of this retrospective study was that the medication dosage could not be adjusted according to the study. Therefore, whether the dosage would influence the association could not be assessed. Also, causal relationship cannot be established due to the nature of an observational study. Thus, a further strictly designed prospective cohort study is needed to confirm our findings. Second, our study only considered subjects being treated with metoprolol, which questions whether our results could be applied to those being treated with other β-blockers. Third, although the intra- and interassay coefficients variation were below 5%, and the blood sample was drawn between 6 AM and 8 AM after overnight fasting at least 10 h, the influence of assay variation or diurnal variation to some extent might still exist. Finally, the patients were recruited from a single university hospital. Patients with serious cardiac events and other macrovascular complications were excluded because of more than one antihypertensive medicine treatment. Therefore, future multi-center prospective studies with participants suffering from macrovascular complications are needed to evidence the association.

## Conclusion

In conclusion, these data reported the TSH-lifting effect of selective β_1_- adrenergic receptor blockers in diabetes with euthyroidism, which might result in higher CVD risks. Our result might also bring another proof about the ameliorated prognosis of hyperthyroidism and thyroid storm from the possible inhibitory effect on secretion of thyroid hormone by β_1_- adrenergic receptor blockers. Further prospective studies conducted on a larger series of patients are required to confirm the association.

## Data Availability

The original contributions presented in the study are included in the article/supplementary material, further inquiries can be directed to the corresponding author.

## Glossary

**95% CIs**: 95% confidence intervals
**ACEI**: angiotensin-converting enzyme inhibitors
**AM**: after midnight
**ANOVA**: analysis of variance
**ARB**: angiotensin receptor blockers
**BMI**: body mass index
**BP**: blood pressure
**CCB**: calcium channel blockers
**Cr**: creatinine
**CVD**: cardiovascular disease
**DBP**: diastolic blood pressure
**DPP-IV inhibitors**: dipeptidyl peptidase-IV inhibitors
**eGFR**: estimated glomerular filtration rate
**fT3**: free triiodothyronine
**fT4**: free thyroxine
**FBG**: fasting blood glucose
**GLP-1 ra**: glucagon likepeptide-1 receptor agonists
**HbA1c**: Hemoglobin A1c
**HDL-C**: high-density lipoprotein cholesterol
**HT**: hypertension
**IQR**: interquartile ranges
**LDL-C**: low-density lipoprotein cholesterol
**ORs**: odds ratios
**RAAS**: renin-angiotensin-aldosterone system
**SBP**: systolic blood pressure
**SD**: standard deviations
**SGLT-2 inhibitors**: sodium-dependent glucose transporters-2 inhibitors
**SPINA-GT**: thyroid’s secretory capacity
**T3**: triiodothyronine
**T4**: thyroxine
**TC**: total cholesterol
**TgAb**: antithyroglobulin antibody
**TGs**: triglycerides
TPOAb: thyroperoxidases antibody
**TSH**: thyroid-stimulating hormone
**TSHI**: Jostel’s TSH index
**TZD**: thiazolidinediones
**WHO**: World Health Organization
